# The Bigger the Better? Center Volume Dependent Effects on Procedural and Functional Outcome in Established Endovascular Stroke Centers

**DOI:** 10.3389/fneur.2022.828528

**Published:** 2022-03-02

**Authors:** Marianne Hahn, Sonja Gröschel, Yasemin Tanyildizi, Marc A. Brockmann, Klaus Gröschel, Timo Uphaus, German Stroke Registry-Endovascular Treatment (GSR-ET) Investigators, A Reich

**Affiliations:** ^1^Department of Neurology, University Medical Center of the Johannes Gutenberg University, Mainz, Germany; ^2^Department of Neuroradiology, University Medical Center of the Johannes Gutenberg University, Mainz, Germany

**Keywords:** stroke, mechanical thrombectomy, GSR-ET, endovascular stroke therapy, procedural volume, center volume

## Abstract

**Background:**

Mechanical thrombectomy (MT) rates for the treatment of acute ischaemic stroke due to large vessel occlusion are steadily increasing, but are delivered in heterogenic settings. We aim to investigate effects of procedural load in centers with established MT-structures by comparing high- vs. low-volume centers with regard to procedural characteristics and functional outcomes.

**Methods:**

Data from 5,379 patients enrolled in the German Stroke Registry Endovascular Treatment (GSR-ET) between June 2015 and December 2019 were compared between three groups: high volume: ≥180 MTs/year, 2,342 patients; medium volume: 135–179 MTs/year, 2,202 patients; low volume: <135 MTs/year, 835 patients. Univariate analysis and multiple linear and logistic regression analyses were performed to identify differences between high- and low-volume centers.

**Results:**

We identified high- vs. low-volume centers to be an independent predictor of shorter intra-hospital (admission to groin puncture: 60 vs. 82 min, β = −26.458; *p* < 0.001) and procedural times (groin puncture to flow restoration: 36 vs. 46.5 min; β = −12.452; *p* < 0.001) after adjusting for clinically relevant factors. Moreover, high-volume centers predicted a shorter duration of hospital stay (8 vs. 9 days; β = −2.901; *p* < 0.001) and favorable medical facility at discharge [transfer to neurorehabilitation facility/home vs. hospital/nursing home/in-house fatality, odds ratio (*OR*) 1.340, *p* = 0.002]. Differences for functional outcome at 90-day follow-up were observed only on univariate level in the subgroup of primarily to MT center admitted patients (mRS 0–2 38.5 vs. 32.8%, *p* = 0.028), but did not persist in multivariate analyses.

**Conclusion:**

Differences in efficiency measured by procedural times call for analysis and optimization of in-house procedural workflows at regularly used but comparatively low procedural volume MT centers.

## Introduction

Mechanical thrombectomy (MT), besides intravenous thrombolysis (IVT), is one of the key elements of acute recanalisation in ischaemic stroke therapy. Evidence for its benefit in functional outcome following acute ischaemic stroke due to large vessel occlusion has been shown in multiple randomized controlled trials (1), so that MT is considered to be the standard of care in these patients. During the past years, proportions of patients with acute ischaemic stroke treated with MT have continuously risen, e.g., from 1.6% in 2012 to 6.5% in 2018 in Germany (2). In addition to existing evidence of clinical efficacy, effectiveness in everyday patient care is now subject to assessment in various analyses of real-life patient data. Analysis of outcomes in routine care is important, since patient cohorts in clinical routine differ from trial cohorts with strict inclusion criteria (3–5). In addition, site-inherent aspects including workflow of the thrombectomy procedure have already been considered to affect the clinical benefit of MT and trial outcomes (6). Thus, there is the need to identify obstacles in clinical routine to allow optimisation to yield as much clinical benefit of MT within our everyday health service as possible. Acute stroke care is delivered in quite heterogenic environments. Disparities in MT use and stroke mortality have been shown to vary depending on regional factors (7) and hospital-specific factors, such as hospital care level, stroke center certification (8, 9), and procedural volume. There is increasing evidence that high annual procedural volume is associated with increased procedural efficiency, better patient outcomes, and decreased mortality (10–13). Analyses of low procedural volume centers within the United States have so far focused on centers performing a yearly case number of <10 (10), <24 (12, 13), or <50 MTs (11). However, these numbers do not reflect the situation in western countries, such as Germany. During 2015–2017, 49.5% (*n* = 148) of all certified stroke centers in Germany offered MT, each performing from 1 to 291 interventions per year. Most MTs (86%) were carried out by 101 over-regional certified stroke centers with a mean MT volume of 79 per year, whereas the other 14% of MTs performed took place at 47 regional certified stroke centers with a mean of 24 interventions per year (14).

Our analysis investigates the effects of high-center volume vs. low-center volume in the German prospective, multicentre Stroke Registry Endovascular Treatment (GSR-ET), enrolling patients with acute ischaemic stroke due to large vessel occlusion undergoing MT. The 25 centers enrolling in GSR-ET are comparatively high in yearly procedural volume with a median of 159 MTs in 2019 (range: 35–300). This allows us to focus on the effects of center volume on established MT-structures in an environment in which these are used on a regular basis. We aimed to, first, assess whether the functional outcome differs in patients treated in high- vs. low-volume centers. Second, we sought to compare procedural times and outcomes to identify potential areas for targeted quality improvement by process optimisation in high- vs. low-volume centers.

## Methods

### Standard Protocol Approval and Data Availability

Study protocols and procedures were conducted in compliance with the Declaration of Helsinki and in accordance with the local ethical guidelines. The GSR-ET was approved by the ethics committee of the Ludwig-Maximilians University (Munich), as the leading center (protocol no. 689-15), and by the local ethics committees. Written informed consent was obtained from all participants (or guardians of participants) in the study. The data supporting the findings of this study are available from the corresponding author on a reasonable request from any qualified investigator.

### Study Population

All patients included in our analysis are part of the GSR-ET, an ongoing academic, independent, prospective, multicentre, observational registry study registered at ClinicalTrials.gov (Identifier: NCT03356392). In this study, 25 certified German stroke centers consecutively enroll patients (1) diagnosed with acute ischaemic stroke due to large vessel occlusion; (2) who intend to be treated with MT; (3) are at least 18 years old; and (4) provide informed consent themselves or by proxy. Baseline demographics, comorbidities, clinical and procedural information, as well as clinical follow-up after 90 days are recorded. More detailed information on the registry's study protocol and variables has recently been published (15).

**Figure 1:**
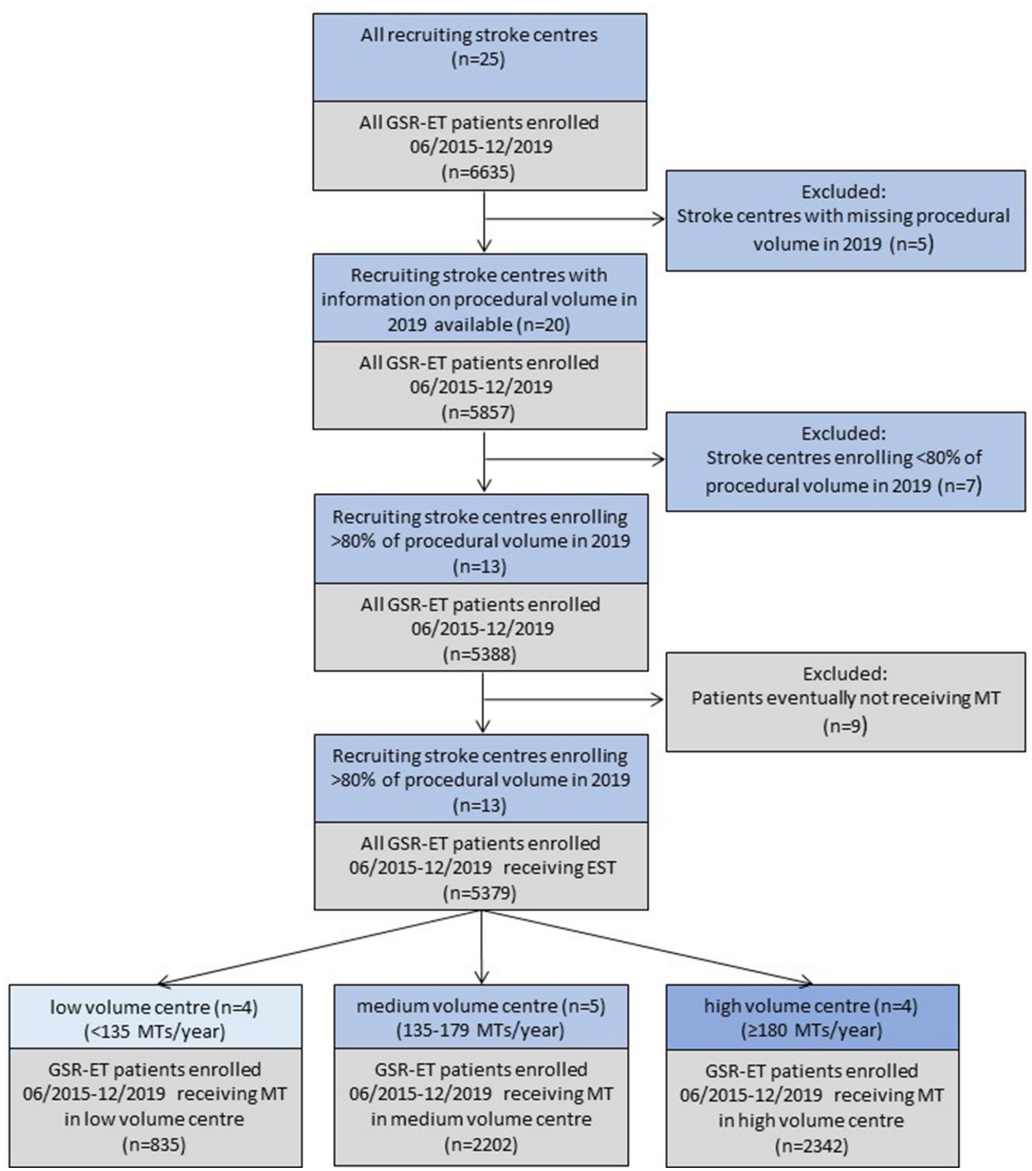
Patient selection and comparison grouping. MT, mechanical thrombectomy; GSR-ET, German Stroke Registry Endovascular Treatment. Colors: blue: GSR-ET enrolling centers and center-specific exclusion criteria; grey: resulting patient cohort and patient-specific exclusion criteria.

The selection criteria workflow for this analysis is depicted in Figure 1. Briefly, from 6,635 total patients who were included in the GSR-ET between July 2015 and December 2019, we included only those patients whose treating center provided information about the absolute number of MT procedures carried out in 2019 (*n* = 5,857). To address recruiting bias and increase generalizability of the findings, we included only patients treated in centers actively recruiting equal or more than 80% of all MT treated patients at their facility in 2019. Patients that eventually did not receive endovascular treatment, but were still enrolled in the register, were excluded. Ultimately, 5,379 patients were included in our analysis.

### Comparison Groups and Outcome Parameters

Centers (*n* = 13) were ordinally ranked and grouped in tertiles by procedural volume in the year 2019, resulting in the low-volume tertile (*n* = 4) being equivalent to <135 MTs per year (range: 35–118 MTs per year) and the high-volume tertile (*n* = 4) being equivalent to at least 180 interventions per year (range: 188–236 MTs per year). Medium-volume centers ranged between 135 and 179 MTs per year (*n* = 5). Outcome parameters were set at procedural level [procedural times, successful recanalization (TICI 2b/3)], at discharge level (length of hospital stay, discharge transfer to neurorehabilitation or home as compared with discharge to hospital/nursing home, or in-house fatality) as well as at a functional level at 90-day follow-up [good functional outcome (modified Rankin scale (mRS) ≤ 2), excellent functional outcome (mRS ≤ 1), and health-related quality of life (EQ5d-index)]. For the calculation of health-related quality of life, self (or proxy) reported answers of the patient to the German language version of EQ-5D-3L, included in the 90-day follow-up interview, were used to calculate an EQ5d-index value. The value attached to each health state validated for the German population by time trade-off was used according to Greiner et al. (16). Deceased patients were imputed with 0.

### Statistical Analysis

Data are presented as mean ± SD, median with interquartile range (IQR) or proportions (categorical variables), if not indicated otherwise. Comparison between high- and low-volume centers on the univariate level was performed by the Chi-square test, Fisher's exact test, Kruskal–Wallis test or ANOVA with Tukey's *post-hoc* test as appropriate. Binominal logistic and linear regression analyses were conducted including variables with *p* < 0.05 in univariate analysis and/or when clinically relevant. The multivariate regression analysis was performed for each outcome parameter on the basis of complete datasets of the analyzed outcomes and predictor variables. The Hosmer–Lemeshow test of goodness of fit was used in these regression models. The Bonferroni correction for multiple testing was applied in univariate comparison of medians with reporting of corrected *p* only. A significant difference was considered for *p* < 0.05. Statistical analyses were performed using SPSS® (Version 26, IBM®, Armonk, NY, USA). For subgroup analyses, we included only patients primarily admitted to the MT center.

## Results

### Baseline Characteristics

In total, 5,379 patients were included in our analysis (median age 76.0 years, 50.5% women), of which 835 were treated at centers with low procedural volume (<135 MTs in 2019), 2,202 received MT at medium-volume centers (135–179 MTs in 2019), and 2,342 patients were treated at high-volume centers (≥180 MTs in 2019). The registry data contained 7,438 missing observations out of 161,370 total observations of relevance for our analysis (4.42%). Baseline characteristics are displayed in Table 1.

**Table 1:** Baseline characteristics of patients.

**Variable**	**Low volume center (<135 MTs/year)**	**Medium volume center (135–179 MTs/year)**	**High volume center (≥180 MTs/year)**	***P*** **-value (low vs. high)**
**(*>**n*** of 5,379 observations available)**				
*N*	835	2,202	2,342	
Age (5,375)	66 (77–83)	65 (76–82)	65 (76–83)	1.000
Female (5,375)	50.4% (421)	50.9% (1,119)	50.1% (1,174)	0.894
Pre-mRS (5,291)	0 (0–1)	0 (0–1)	0 (0–1)	0.210
NIHSS on admission (5,300)	16 (11–20)	10 (15–18)	15 (9–19)	**0.003**
IV-thrombolysis (5,337)	44.9% (375)	54.0% (1,181)	49.0% (1,133)	**0.043**
Primary admission at MT site (5,112)	74.7% (620)	62.9 % (1,230)	42.1% (979)	**<0.001**
**Cardiovascular risk factors**				
Arterial hypertension (5,328)	82.7% (689)	78.1% (1,692)	75.7% (1,764)	**<0.001**
Diabetes mellitus (5,320)	28.8% (240)	20.1% (434)	21.3% (496)	**<0.001**
Dyslipidaemia (5,310)	42.3% (351)	38.7% (833)	40.3% (939)	0.301
Atrial fibrillation (5,323)	45.2% (376)	43.2% (933)	41.0% (955)	**0.031**
Smoker (current) (4,774)	15.0% (124)	14.4% (276)	16.0% (324)	0.350
**Location of occlusion (5,297)**				
Carotid artery	22.5% (187)	23.8% (508)	28.8% (672)	**<0.001**
ACA	2.2% (18)	2.2% (47)	2.8% (65)	0.338
MCA M1	59.5% (494)	55.1% (1,176)	47.5% (1,108)	**<0.001**
MCA M2	18.7% (155)	20.0% (426)	21.4% (500)	0.091
PCA	2.4% (20)	3.3% (71)	1.8% (43)	0.317
VB	9.6% (80)	12.1% (258)	10.4% (243)	0.525

*Data are presented as percentage (absolute number) except for age, pre-mRS, NIHSS on admission: median [interquartile range (IQR)]*.

*MT(s), mechanical thrombectomy(s); Pre-mRS, modified Rankin Scale before admission; NIHSS, National Institutes of Health Stroke Scale; ACA, Anterior cerebral artery; MCA M1, Middle cerebral artery M1 segment; MCA M2, Middle cerebral artery m2 segment; PCA, Posterior cerebral artery; VB, vertebrobasilar*.

*Signficiant p-values < 0.05 are displayed in bold*.

Significant differences between patients treated at high- vs. low-volume centers were observed for cardiovascular risk factors (arterial hypertension, diabetes mellitus, and atrial fibrillation), which were more frequent in low-volume centers. Low-volume centers treated patients with slightly higher National Institutes of Health Stroke Scale (NIHSS) on admission, reported more middle cerebral artery occlusions in the M1 segment, and less carotid artery occlusions. Patients treated at high-volume centers received IVT more frequently and were much more likely to have been transferred to the center for MT following primary admission elsewhere.

### Univariate Analysis of Outcome Parameters

#### Procedural Outcome Parameters

**Figure 2:**
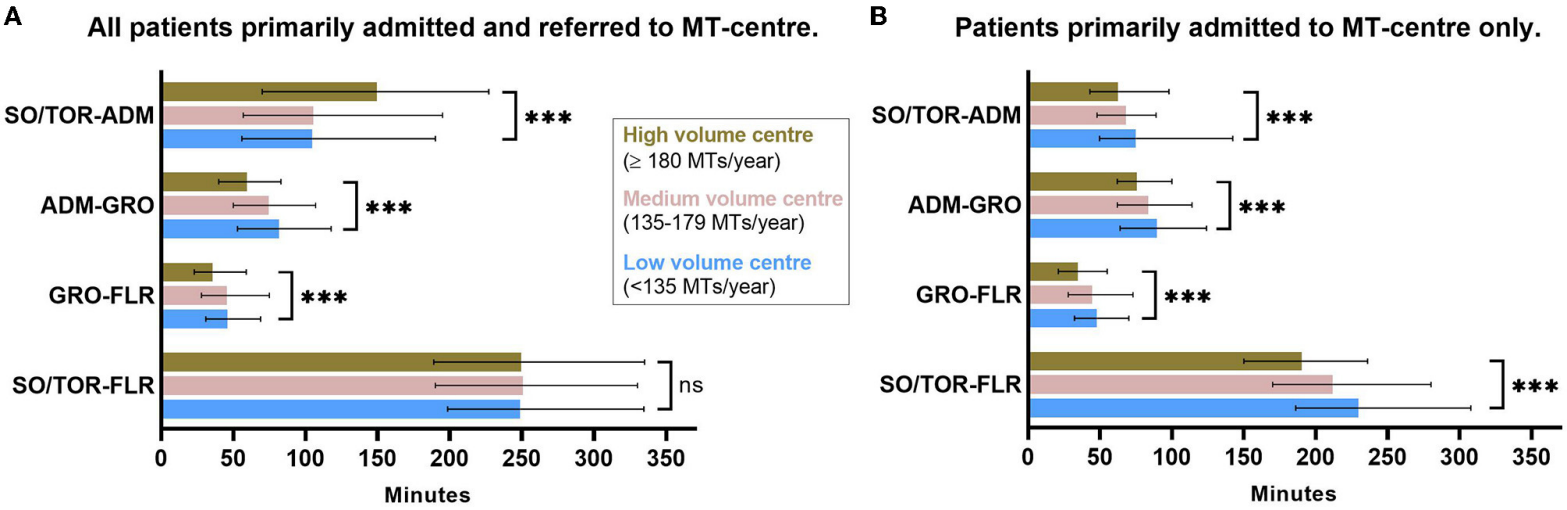
Procedural times depending on center volume tertile. **(A)** Study cohort including all patients. Longer pre-hospital and shorter intra-hospital procedural times in high- vs. low-volume centers. No difference in overall procedural time. **(B)** Subgroup analysis: only patients primarily admitted to MT center. Shorter pre-hospital, intra-hospital, and overall procedural times in high- vs. low-volume centers. Displayed: median with interquartile range (IQR). ***Bonferroni-corrected *p* ≤ 0.001 in univariate comparison high- vs. low-volume center resulting from the pairwise Kruskal–Wallis test. SO/TOR, symptom onset/time of recognition; ADM, admission; GRO, groin puncture; FLR, flow restoration.

In univariate analysis of procedural parameters (Table 2), we observed no significant difference in successful recanalisation rates (TICI 2b-3) between high and low volume centers. Number of passages were higher and the use of general anesthesia was lower in high-volume centers. With regard to procedural times, much longer times from symptom onset/time of recognition to hospital admission were assessed in high-volume centers [150 (IQR: 70–227) vs. 105 (IQR: 56–190) min; *p* < 0.001], while admission to groin puncture [60 (IQR: 40–83) vs. 82 (IQR: 53–117.75) min; *p* < 0.001] as well as groin puncture to flow restoration times [36 (IQR: 23–59) vs. 46.5 (IQR 31–69) min; *p* < 0.001] were significantly shorter in high-volume centers compared with low-volume centers (as shown in Figure 2A). On univariate level, overall symptom onset/time of recognition to flow restoration times did not differ significantly. Duration of hospital stay was found to be shorter in high-volume centers [8 (IQR: 5–12) vs. 9 (IQR: 6–16) days; *p* < 0.001].

**Table 2:** Univariate analysis of procedural and functional outcomes of patients treated in “low-volume centers” compared with “medium-” and “high-volume centers.”

**Variable**	**Low volume center (<135 MTs/year)**	**Medium volume center (135–179 MTs/year)**	**High volume center (≥180 MTs/year)**	***P*** **-value (low vs. high)**
**(***n*** of 5,379 observations available)**			
**Procedural parameters and outcomes**				
Any general anesthesia (5,253)	80.8% (665)	68.6% (1,465)	72.6% (1,667)	**<0.001**
No of passages (4,920)	2.03 ± 1.86	2.22 ± 1.81	2.24 ± 1.85	**0.016**
Successful rec. (5,281)	85.7% (709)	84.4% (1,809)	83.1% (1,919)	0.075
**Procedural times**				
SO/TOR-ADM (minutes) (4,840)	105 (56–190)	106 (57–195)	150 (70–227)	**<0.001**
ADM-GRO (minutes) (5,089)	82 (53–117.75)	75 (50–107)	60 (40–83)	**<0.001**
GRO-FLR (minutes) (4,535)	46.5 (31–69)	46 (28–75)	36 (23–59)	**<0.001**
SO/TOR-FLR (minutes) (4,151)	249 (198,5–334,5)	251 (190–330)	250 (189–335)	1.000
Length of stay (days) (5,347)	9 (6–16)	9 (6–14)	8 (5–12)	**<0.001**
**Functional outcomes**				
DC-Transfer home / neurorehabilitation (5,341)	62.5% (520)	67.2% (1,464)	67.8% (1,581)	**0.005**
90 d excellent outcome (mRS 0–1) (4,704)	23.1% (162)	25.2% (495)	23.5% (481)	0.829
90 d good outcome (mRS 0–2) (4,704)	33.1% (232)	36.1% (707)	35.8% (731)	0.207
90 d lethal outcome	29.9% (209)	29.9 (586)	29.5 (603)	0.864
90 d EQ5d-3L-Index (3,981)	0.32 (0.00–0.76)	0.26 (0.00–0.76)	0.36 (0.00–0.76)	1.000

*Data are presented as percentage (absolute number) except for No of passages: mean ± SD; procedural times; length of stay; 90 days EQ5d-Index: median (IQR)*.

*MT(s), mechanical thrombectomy(s); Successful rec., Successful recanalization (TICI 2b-3); SO/TOR, symptom onset/time of recognition; ADM, admission; GRO, groin puncture; FLR, flow restoration; DC, discharge; mRS, modified Rankin Scale; 90 d, at 90 day follow-up*.

*Signficiant p-values < 0.05 are displayed in bold*.

#### Functional Outcome Parameters

**Figure 3:**
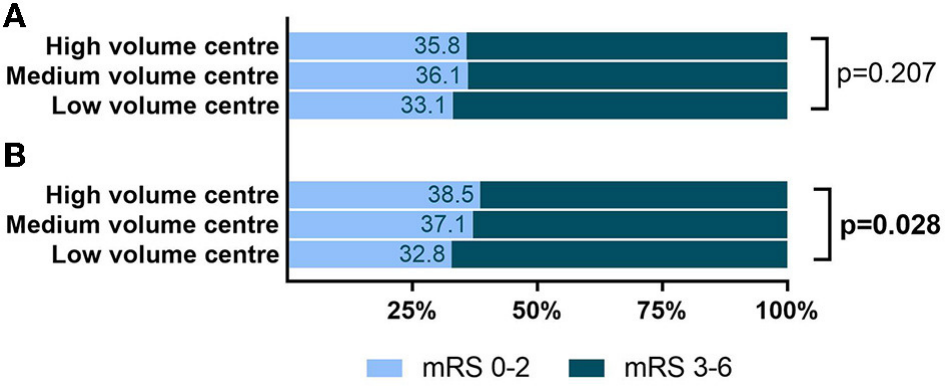
Functional outcome at 90-day follow-up depending on center volume tertile. **(A)** Study cohort including all patients. No significant difference in good functional outcome in high- vs. low-volume center. **(B)** Subgroup analysis: only patients primarily admitted to MT center. Higher share of good functional outcome (mRS 0–2) at 90-day follow-up in high- vs. low-volume centers. Displayed: absolute share of mRS-based good functional outcome. Staded are *p*-values of univariate comparison high- vs. low-volume center resulting from the chi-square test. mRS, modified Rankin scale.

Good functional outcomes at discharge, represented by discharge transfer to a neurorehabilitation facility or home were observed significantly more often at high-volume centers (57.8 vs. 62.5%; *p* = 0.005). On follow-up at 90 days post stroke, neither mRS-based functional outcome parameters nor health-related quality of life in terms of EQ5d index differed between patients receiving MT at high- versus low-volume centers (Figure 3A).

## Multivariate Analysis of Outcome Parameters

With regard to procedural times, MT in high- vs. low-volume centers did not independently predict pre-hospital time from symptom onset/time of recognition to admission, when correcting for baseline characteristics, IVT, and primary admission at intervention hospital (Table 3). In contrast, MT in high- vs. low-volume centers was an independent predictor of shorter admission to groin puncture and groin puncture to flow restoration times as well as for shorter overall symptom onset/time of recognition to flow restoration times. Moreover, correction for the above-mentioned predictors plus the use of anesthesia, recanalisation outcomes, and intra-hospital complications left high- vs. low-volume centers an independent predictor of shorter duration of hospital stay. Favorable functional outcomes were independently predicted by MT in high- vs. low-volume centers only at discharge level [odds ratio (*OR*) 1.340; 95% *CI*: 1.112–1.614; *p* = 0.002 for discharge transfer to neurorehabilitation facility or home]. At 90-day follow-up, MT in high- vs. low-volume centers was not significantly associated with mRS-based functional outcome or health-related quality of life (EQ5d-index).

**Table 3:** Odds ratios/regression coefficients for “high-” vs. “low-volume center” variable resulting from multiple logistic/linear regression analysis for procedural and functional outcome variables adjusting for baseline characteristics.

**Outcome variable**	**OR “high vs. low volume center”**	**Regression coefficient “high vs. low volume center”**	**95% CI**	***P*** **-value**
**Procedural times and outcomes**				
SO/TOR-ADM (minutes)		−11.375	−29.848 - 7.098	0.227
ADM-GRO (minutes)		−26.458	−39.274 - −12.641	**<0.001**
GRO-FLR (minutes)		−12.452	−18.980 - −5.923	**<0.001**
SO/TOR-FLR (minutes)		−43.789	−68.542 - −19.036	**0.001**
Length of stay (days)		−2.901	−3.686 - −2.115	**<0.001**
No of passages		0.162	0.010 - 0.315	**0.037**
Successful rec.	0.862		0.681 - 1.091	0.217
**Functional outcomes**				
DC-Transfer home/neurorehabilitation	1.340		1.112 - 1.614	**0.002**
90 d excellent outcome (mRS 0–1)	0.955		0.745 - 1.223	0.715
90 d good outcome (mRS 0–2)	1.034		0.824 - 1.297	0.774
90 d lethal outcome	1.050		0.839 - 1.315	0.668
90 d EQ5d-3L-Index		−0.004	−0.033 - 0.024	0.780

*Corrected for age, sex, mRS before admission, NIHSS on admission, CVRFs: arterial hypertension, diabetes mellitus, atrial fibrillation, location of occlusion: carotid artery, middle cerebral artery M1, IV thrombolysis, and whether patient's primary admission was at intervention hospital: except length of stay: additionally corrected for general anesthesia, successful recanalization, adverse events during hospital stay: any, dissection/perforation, clot migration/embolization, intracranial hemorrhage, vasospasm, malignant media infarction, and other*.

*SO/TOR, symptom onset/time of recognition; ADM, admission; GRO, groin puncture; FLR, flow restoration; DC, discharge; mRS, modified Rankin Scale; 90 d, at 90 day follow-up; OR, odds ratio*.

*Signficiant p-values < 0.05 are displayed in bold*.

## Subgroup Analysis of Patients Primarily Admitted to MT Center

Within the 2,829 patients primarily admitted to MT center, univariate comparison of procedural outcome parameters (Supplementary Table 1) showed shorter pre-hospital [symptom onset/time of recognition to admission: 63 (43–98) vs. 75 (51–141) min; *p* < 0.001], as well as shorter intra-hospital times [admission to groin puncture: 76 (62–100) vs. 90 (64–124) min; *p* < 0.001 and groin puncture to flow restoration: 35 (21–55) vs. 48 (32.25–70) min; *p* < 0.001] in high- vs. low-volume centers (as shown in Figure 2B). Moreover, in univariate comparison, we noted better functional outcome parameters in high- vs. low-volume centers at discharge level (transfer to a neurorehabilitation facility or home: 71.4 vs. 62.7%; *p* < 0.001) as well as for the mRS-based functional outcome on follow-up at 90 days (mRS 0–2: 38.5% vs. 32.8%; *p* = 0.028, Figure 3B). In multiple regression analyses adjusting for covariates (Supplementary Table 2), high- vs. low-volume was a significant independent predictor for shorter overall procedural time, intra-hospital (groin puncture to flow restoration), and pre-hospital time. With regard to the functional outcome, high- vs. low-volume center was an independent predictor of favorable functional outcome only at discharge level, while center volume did not independently predict the functional outcome at 90 days follow-up after adjustment.

## Discussion

With increasing numbers of MT procedures in the treatment of acute ischaemic stroke due to large vessel occlusion, evaluation of effectiveness in everyday patient care is necessary in addition to early controlled trials proving therapeutic efficacy. We here provide evidence that, in established endovascular stroke centers, procedural times of the acute recanalization procedure as well as the duration of hospital stay, reflecting organizational workflow in the subacute phase after MT, are influenced by center volume-specific effects. Whereas, no independent predictive capacity of center volume on the functional outcome or health-related quality of life at 90-day follow-up was observed. Despite better functional outcome in high- vs. low-volume centers on univariate level in the subgroup of patients primarily admitted at MT center, the predictive capacity of procedural load on functional outcome did not persist after correction for confounders in the multivariate analysis in this subgroup either. This indicates an effective MT procedure, also in the comparatively low-volume centers.

Procedural volume and experience have been suspected to influence outcome parameters on different levels. We show that a higher number of annual procedures are associated with faster in-house procedural times (admission to groin puncture as well as groin puncture to flow restoration). This is consistent with an analysis of Gupta et al. (11), which was performed during MT-routine establishment, and compared centers performing more than the median of 50 MTs/year to lower volume centers. We add new evidence, showing that the scope of this learning curve exceeds early MT structure establishment and persists in regularly used MT environments with substantial procedural volume—all sites in our analysis performed at least 35 MTs/year with a median of 146 yearly procedures. Intra-hospital time delay, which we observed in low-volume centers have recently been shown to be particularly associated with loss of healthy life—years (17) and should be addressed by in-depth analysis and optimization of the local MT workflow. Overall time from symptom onset/time of recognition to flow restoration appeared not to be different in high- vs. low-volume centers. However, in the subgroup analysis and after adjusting for whether a patient was primarily admitted to another hospital, higher-volume center was an independent predictor for a shorter overall time until flow restoration. Hence, high-volume MT centers were able to compensate for lower primary admission rates (42.1 vs. 74.7%, *p* < 0.001) and delays due to transfer time with higher efficiency and faster in-house procedural times. Delay due to transfer from the primary admission site to MT facility and resulting lower chance of good outcome has been described by several studies (18, 19). Especially high-volume centers with high transfer rates might therefore benefit from an analysis of how interaction and processes with referring institutions could possibly be organized more efficiently.

We found that the functional outcome as measured by the mRS and health-related quality of life at 90 days was not influenced by high- vs. low-procedural volume in our dataset. Although the subgroup analysis of only primarily to MT facility admitted patients suggested better long-term functional outcome on univariate level, also in this group, when adjusting for differences in baseline characteristics, center volume did not independently predict favorable functional outcome. This observation is in contrast with several studies demonstrating better functional and procedural outcomes in high-volume centers. However, low-volume centers analyzed before had offered as few as 10 (10), 24 (12, 13), or 50 (11) MTs/year and were derived from datasets with a comparatively low proportion of MT sites performing substantial procedural volume in relation to the currently observed distribution of procedural volume over all MT facilities. Acute stroke care in western countries, e.g., Germany, is today shaped by supraregional, firmly established and regularly used MT facilities that passed the early learning curve and perform 86% of all MTs with a median of 79 annual procedures (14). A recent analysis of the development of MT skills in the process of MT establishment described a correlation of reperfusion rates and functional outcome with increasing cumulative MT case volumes comparing a facility's initial MT procedures with the outcomes after having performed more than 150 total cases (20). We augment the analysis by studying a cohort of centers that already perform MT procedures on a regular basis, which might have balanced the unfavorable effect of low procedural volume on the functional outcome measures seen before. This is supported by the absence of difference in reperfusion rates in our data, as a measure of procedural skills, in high- vs. low-volume centers. Another explanation for the lack of effect of procedural volume on the mRS-based outcome or health-related quality of life in our data might be due to the similar overall procedural times in high- and low-volume centers. Moreover, high-volume centers have already been treating more complex cases with significantly more occlusions of the carotid artery, and yet achieving the same rates of reperfusion and good functional outcome. Nonetheless and in agreement with Saber et al. (13), we found differences between the functional outcome at discharge, measured by transfer to neurorehabilitation facilities or home (vs. hospital/nursing home/in-house fatality), which was more frequent in high-volume centers. However, they did not have long-term functional outcome parameters available to evaluate these effects for sustainability. This finding might be confounded by improved organizational structures or network collaborations in high-volume centers with regard to transfer institutions so that we consider functional outcome at 3 months follow-up as a more robust outcome parameter.

Interestingly, looking at the overall duration of hospital stay, processes in low-volume MT centers might be optimized with regard to the patient management following acute stroke care. We found significantly longer durations of hospital stay in low-volume centers, notably after adjustment for patient characteristics and intra-hospital MT-associated complications. This might partly result from a more routine workflow, since the standardized care resulting in stroke center certification has been shown to be associated with a tendency of shorter length of stay in Michigan, United States (21). An in-depth analysis of factors prolonging intra-hospital stay within less practiced institutions might therefore result in both a prognostically favorable (22) patient's early transfer to a neurorehabilitation, thereby decreasing stroke-related disabilities, and a more efficient allocation of economic and healthcare resources.

Despite the strengths of analyzing >5,000 MT procedures, we are aware of study limitations with regard to the nature of prospective registry data, which are prone to recruitment bias and missing data, limiting the generalizability of our findings. In addition, our analysis includes patients treated from 2015 to 2019, therefore individual centers might have increased or decreased their annual volume due to structural in-house changes or the local network of facilities offering acute stroke care. Nevertheless, comparing the low-volume tertile to the high-volume tertile increases the stability of comparison groups, compensating for slight changes in relative annual procedural volume over time. By analyzing a large and complex, nationwide cohort of patients treated with MT, the wide extent of data acquired through the GSR-ET database allows a broad and robust analysis of procedural outcome and efficiency as well as multiple functional outcome parameters and extensive adjustment for potential confounders, such as pre-hospital functional status, comorbidities, and stroke severity. Using up-to-date registry data from 2015 to 2019 allows us to focus on MT structures in a time of rising procedural volume rather than during the establishment of previously non-existent organizational structures. Since the GSR-ET enrolling centers in general have comparatively high procedural volume, our analysis is well-suited to assess MT structures in established environments but lacks transferability to regional stroke centers, yet, accounting for only 14% of all MTs.

## Conclusion

Knowledge about the effects of site-specific factors is a crucial factor for improving real-life patient care by health service policymaking with regard to the resource allocation in acute stroke care in a heterogenic environment of care providers. Our findings have direct implications on the debate surrounding the optimal organization of acute stroke care, which meanders between calls for centralization with a maximum degree of expertise and widespread decentralized, accessible smaller MT sites (10, 23). We found that in established and regularly used MT facilities, procedural volume did not independently predict long-term functional outcomes. However, our findings indicate that the learning curve with regard to MT efficiency is not limited to the period of MT structure establishment. Differences in procedural times call for focused evaluation of the MT process to optimise in-house MT procedures in established but comparatively low-volume MT centers. We report time delay for the time from admission to groin puncture as well as time from groin puncture to flow restoration. Targeted on-site analysis of all sequential components involved in the MT procedure might reveal unsteady flows or shortages that can be addressed and enable achievement of faster procedural times in low-volume centers, as would naturally occur with higher procedural volume. On the other hand, our findings advocate avoiding a local redundancy in services to allow for a naturally occurring increase of efficiency, as observed for centers with high procedural volume. Further research is needed concerning mechanisms of center volume-dependent differences in outcome parameters. In addition, analysis of more center-specific factors could add valuable insights into what can help to make evidence-based medicine universally available, still allowing for individual differences within the settings of our healthcare services.

## Data Availability Statement

The data supporting the findings of this study are available from the corresponding author on a reasonable request from any qualified investigator.

## Ethics Statement

Study protocols and procedures were conducted in compliance with the Declaration of Helsinki and in accordance with the local ethical guidelines. The GSR-ET was approved by the ethics committee of the Ludwig-Maximilians University (Munich), as the leading center (protocol no. 689–15), and by the local ethics committees. Written informed consent was obtained from all participants (or guardians of participants) in the study.

## German Stroke Registry-Endovascular Treatment (GSR-ET) Investigators

GSR-ET investigators: A Reich, O Nikoubashman, J Röther, B Eckert, M Braun, G F Hamann, E Siebert, C H Nolte, G Bohner, R M Eckert, J Borggrefe, P Schellinger, J Berrouschot, A Bormann, C Kraemer, H Leischner, M Petersen, F Stögbauer, T Boeck-Behrens, S Wunderlich, A Ludolph, K H Henn, C Gerloff, J Fiehler, G Thomalla, A Alegiani, J H Schäfer, F Keil, S Tiedt, L Kellert, C Trumm, U Ernemannn, S Poli, J Liman, M Ernst, K Gröschel, T Uphaus.

## Author Contributions

KG and TU designed and conceptualized the study. TU and MH contributed to data acquisition. MH performed statistical analyses with support from TU and drafted the manuscript for intellectual content. KG, MH, and TU interpreted the data. KG, MB, SG, TU, and YT revised the manuscript. All authors contributed to the article and approved the submitted version.

## Conflict of Interest

TU reports personal fees from Merck Serono and Pfizer, grants from Else Kröner-Fresenius Stiftung. KG reports personal fees and/or non-financial support from Bayer, Boehringer Ingelheim, Bristol-Meyers Squibb, Daiichi Sankyo, and Pfizer. MH reports personal fees from Bristol-Meyers Squibb, outside of the submitted work. The remaining authors declare that the research was conducted in the absence of any commercial or financial relationships that could be construed as a potential conflict of interest.

## Publisher's Note

All claims expressed in this article are solely those of the authors and do not necessarily represent those of their affiliated organizations, or those of the publisher, the editors and the reviewers. Any product that may be evaluated in this article, or claim that may be made by its manufacturer, is not guaranteed or endorsed by the publisher.

## Supplementary Material

Supplementary Table 1 Subgroup analysis: procedural and functional outcomes of patients primarily admitted to MT center treated in “low volume centers” compared with “medium-” and “high-volume centers”.

Supplementary Table 2 Subgroup analysis: odds ratios (*OR*)/regression coefficients for “high-” vs. “low-volume center” variable resulting from multiple logistic/linear regression analysis for procedural and functional outcome variables in patients primarily admitted to the mechanical thrombectomy (MT) center.
