# Supplementary material for: The Bigger the Better? Center Volume Dependent Effects on Procedural and Functional Outcome in Established Endovascular Stroke Centers
Source: Front Neurol. 2022 Mar 2;13:828528. doi: 10.3389/fneur.2022.828528 (PMC8925986; doi:10.3389/fneur.2022.828528)
Supplement: Supplementary Table 1 — Subgroup analysis: procedural and functional outcomes of patients primarily admitted to MT center treated in “low volume centers” compared with “medium-” and “high-volume centers”. [file Table_1.DOCX]

**SUPPLEMENTAL MATERIAL**

**Table S1. Subgroup analysis:** Procedural and functional outcomes of patients primarily admitted to MT centre treated in ‘low volume centres’ compared to ‘medium’ and ‘high volume centres’.

| Variable  (n of 2829 observations available) | Low volume centre (<135 MTs/year, n=620) | Medium volume centre (135-179 MTs/year, n=1230) | High volume centre (≥180 MTs/year, n=979) | p value  (low vs. high) |
| --- | --- | --- | --- | --- |
| *Procedural parameters and outcomes* | | | | |
| Any general anaesthesia (2760) | 80.0% (489) | 67.5% (805) | 77.8% (745) | 0.303 |
| No of passages (2585) | 1.93 ± 1.47 | 2.18 ± 1.79 | 2.17 ± 1.72 | **<0.001** |
| Successful rec. (2779) | 85.3% (523) | 84.2% (1011) | 82.9% (800) | 0.204 |
| *Procedural Times* | | | | |
| SO/TOR-ADM (minutes) (2524) | 75 (51-141) | 68.5 (48-109) | 63 (43-98) | **<0.001** |
| ADM-GRO (minutes) (2655) | 90 (64-124) | 84 (62-114) | 76 (62-100) | **<0.001** |
| GRO-FLR (minutes) (2351) | 48 (32.25-70) | 45 (27.5-73) | 35 (21-55) | **<0.001** |
| SO/TOR-FLR (minutes) (2139) | 230 (186-305.75) | 212 (170-280) | 190.5 (150-245) | **<0.001** |
| Length of stay (days) (2816) | 10 (6-16.5) | 9 (6-14) | 8 (5-13) | **<0.001** |
| *Functional outcomes* | | | | |
| DC-Transfer home / neurorehabilitation (2813) | 62.7% (387) | 68.4% (834) | 71.4% (697) | **<0.001** |
| 90d excellent outcome (mRS 0-1) (2531) | 22.9% (128) | 26.1% (289) | 27.7% (240) | **0.046** |
| 90d good outcome (mRS 0-2) (2531) | 32.8% (183) | 37.1% (410) | 38.5% (335) | **0.028** |
| 90d lethal outcome (2531) | 30.1% (168) | 29.4 (325) | 29.4 (255) | 0.779 |
| 90d EQ5d-3L-Index (2114) | 0.33 (0.00-0.76) | 0.26 (0.00-0.76) | 0.44 (0.00-0.76) | 1.000 |

Table legend: Data are presented as percentage (absolute number) except for No of passages: mean ± SD; Procedural Times; Length of stay; 90d EQ5d-Index: median (IQR). Abbreviations: MT(s): mechanical thrombectomy(s); Successful rec.: Successful recanalization (TICI 2b-3); SO: symptom onset; TOR: time of recognition; ADM: admission; GRO: groin puncture; FLR: flow restoration; DC: discharge; mRS: modified Rankin Scale; 90d: at 90 day follow-up.

**Table S2. Subgroup analysis:** Odds Ratios / Regression coefficients for ‘high’ vs. ‘low volume centre’ variable resulting from multiple logistic / linear regression analysis for procedural and functional outcome variables in patients primarily admitted to MT centre.

| Outcome Variable | OR ‘high vs. low volume centre’ | Regression Coefficient ‘high vs. low volume centre’ | 95% CI | p-value |
| --- | --- | --- | --- | --- |
| *Procedural Times* | | | | |
| SO/TOR-ADM (minutes) |  | -26.357 | -51.300- -1.413 | **0.038** |
| ADM-GRO (minutes) |  | -15.245 | -31.471-0.980 | 0.066 |
| GRO-FLR (minutes) |  | -13.465 | -22.232- -4.699 | **0.003** |
| SO/TOR-FLR (minutes) |  | -55.342 | -89.337- -21.347 | **0.001** |
| Length of stay (days) |  | -3.113 | -4.162- -2.064 | **<0.001** |
| No of passages |  | 0.231 | 0.058-0.405 | **0.009** |
| Successful rec. | 0.895 |  | 0.670-1.196 | 0.452 |
| *Functional outcomes* | | | | |
| DC-Transfer home/neurorehabilitation | 1.463 |  | 1.155-1.854 | **0.002** |
| 90d excellent outcome (mRS 0-1) | 1.148 |  | 0.854-1.542 | 0.360 |
| 90d good outcome (mRS 0-2) | 1.152 |  | 0.876-1.516 | 0.311 |
| 90d lethal outcome | 1.061 |  | 0.810-1.391 | 0.667 |
| 90d EQ5d-3L-Index |  | -0.002 | -0.036-0.033 | 0.931 |

Table legend: Corrected for age, sex, mRS before admission, NIHSS on admission, CVRFs: Arterial hypertension, Diabetes mellitus, Atrial fibrillation, Location of occlusion: Carotid artery, Middle cerebral artery M1, Middle cerebral artery M2 and IV thrombolysis, except length of stay: additionally corrected for general anaesthesia, successful recanalization, adverse events during hospital stay: any, dissection/perforation, clot migration/embolization, intracranial haemorrhage, vasospasm, malignant media infarction, other. Abbreviations: SO: Symptom onset; TOR: Time of recognition; ADM: Admission; GRO: Groin puncture; FLR: Flow restoration; DC: discharge; mRS: modified Rankin Scale; 90d: at 90 day follow-up, OR: odds ratio.
